# The Character and Treatment of Frost-bite

**Published:** 1918-02

**Authors:** 


***The Character and Treatment of Frost-bite.*** By H. E. MUNROE, Major, C. A. M. C. Abs. from the *Brit. Med. Jour.*, Déc. 23, 1915.

M. believes that prophylaxis requires (1) that men in the trenches should wear loose, water-tight boots, (2) that they should be provided daily with dry woollen socks, (3) that they should move their feet freely ànd so maintain the circulation.

He recommends by way of treatment that every available means be employed to avoid excessive reaction. He argues as follows: " Left to nature the partially devitalized cells become destroyed by the sudden engorgement of the part. The more severe the frost-bite the more edema is present and the more pain is complained of. If the patient is seen while the part is still frozen, the circulation should be restored by placing the limb in ice-cold water, or by using gentle friction, with snow when available. The part may be covered with gauze or with absorbant cotton, which should be frequently saturated with an evaporating lotion. I use a lotion composed of liq. plumbi subacet. 1 dr., spt. rectif. 3 dr., water 1 oz. When blebs form, they should be punctured. The application of evaporating lotion is continued for the first 24 to 36 hours, until the swelling begins to subside or there is an indication of a cyanotic or shrunken appearance in the toes or fingers, if the hand is involved. Hot boracic fomentations should be employed in order to overcome the reactive vaso-motor constriction and consequent anemia of the parts. This treatment is kept up for 24 hours, or until the cyanotic or shrunken appearance disappears or gangrene has definitely formed ". He believes that amputation should not be performed until a line of demarcation is evident, and recommends the use, in the meantime, of a powder of boracic acid and charcoal if any moisture or odor is present.

